# Factors influencing exotic species richness in Argentina’s national parks

**DOI:** 10.7717/peerj.5514

**Published:** 2018-09-04

**Authors:** Mariela G. Gantchoff, Clay M. Wilton, Jerrold L. Belant

**Affiliations:** 1 Forest and Wildlife Research Center, Mississippi State University, MS, United States of America; 2 Michigan Natural Features Inventory, Lansing, MI, United States of America

**Keywords:** Exotic mammals, Exotic plants, Biological invasions, Non-native species, Latin America, Exotic birds, Argentina, Protected area, Alien species

## Abstract

Exotic species introductions are a global phenomenon and protected areas are susceptible to them. Understanding the drivers of exotic species richness is vital for prioritizing natural resource management, particularly in developing countries with limited resources. We analyzed the influence of coarse resolution factors on exotic species richness (plants, mammals, and birds) in Argentina’s National Parks System. We collected data on native species richness, year of park formation, park area, region, elevation range, number of rivers crossing area boundaries, roads entering area, mean annual rainfall, mean annual temperature, mean annual number of visitors, and Human Influence Index within and surrounding each park. We compiled 1,688 exotic records in 36 protected areas: 83% plants and 17% animals (9.5% mammals, 5.5% birds, 1.5% fishes, 0% amphibians, 0% reptiles). The five parks with the most exotic species (all taxa combined) were in north Patagonia. Exotic grasses were the most common exotic plants, and within animals, lagomorphs and feral ungulates were remarkably widespread. Exotic plant richness was mostly influenced by temperature and native plant richness, while exotic mammal and bird richness was driven mostly by anthropogenic variables, with models explaining 36–45% of data deviance. Most variables that positively influenced exotic taxa were indirectly related to an increase in spatial heterogeneity (natural or anthropogenic), suggesting greater niche space variability as facilitators of exotic richness increase. Additional data are needed to further investigate the patterns and mechanisms of exotic species richness in protected areas, which will help to prioritize the greatest needs of monitoring and management.

## Introduction

Creating protected areas is one of the most important strategies for biodiversity conservation (Noss, 1996; Bruner et al., 2001). However, policy and management in and around protected areas are often ineffective (Pressey, Visconti & Ferraro, 2015), and anthropogenic disturbances to protected areas are widespread and common (Macdonald et al., 1989; Brashares, Arcese & Sam, 2001; Liu et al., 2001; Barber et al., 2011; Laurance et al., 2012). Exotic species occur in most ecosystems around the globe (Pyšek & Richardson, 2010), and their impact on protected areas is amplified because of the role of these areas in preserving biological diversity and maintaining essential services for many communities (Foxcroft, Pyšek & Richardson, 2014). Human activities adjacent to protected areas can have numerous negative consequences, including exotic species introductions (e.g.,  Pauchard & Villarroel, 2002) and landscape fragmentation, further isolating protected areas and increasing their susceptibility to disturbance (Spear et al., 2013). Protected area vulnerability is particularly important in developing countries with limited resources to implement effective protection (Bonham, Sacayon & Tzi, 2008).

Research on exotic species has described many aspects of their occurrence, establishment, spread, and impacts (Catford, Jansson & Nilsson, 2009; Lowry et al., 2012), which are in turn related to different branches of ecological theory. In particular, community ecology states that for an introduced species to establish, grow, and spread in a new area, it must encounter the right combination of environmental conditions, resources, and enemies in that invaded community (Lodge, 1993; Chesson, 2000); also termed a “niche opportunity” (Shea & Chesson, 2002; Catford, Jansson & Nilsson, 2009). Several hypotheses have been developed that are related to all or portions of the niche opportunity concept. The community richness hypothesis (Elton, 1958) states that the process of exotic species establishment is affected by the number of native species in the community; a greater number of native species should collectively offer more potential negative interactions, such as competition or predation, to the introduced species. Alternatively, the rich get richer hypothesis states that areas of higher native species diversity also support a high number of exotic species through increased resource availability and diversity (Stohlgren, Barnett & Kartesz, 2003). The environmental hypothesis states that habitats with high environmental variability and productivity contain a diverse array of niches that can host a greater number of exotic species (Melbourne et al., 2007; Catford, Jansson & Nilsson, 2009). Additionally, the human disturbance hypothesis states that disturbance can act as a facilitating agent, decreasing interspecific competition or predation in the invaded community (Elton, 1958; Hobbs & Huenneke, 1992). Evidence of human disturbance benefiting invaders is common in many terrestrial and aquatic communities (Johnson, Olden & Vander Zanden, 2008; Kalwij, Robertson & Van Rensburg, 2008; Crooks, Chang & Ruiz, 2011).

The drivers of exotic species richness can differ from drivers of invasive species richness (i.e., species that cause negative ecological or socioeconomic effects), and research on biological invasions should consider investigating all exotic species, not only those known to have expanded and caused negative impacts (Spear et al., 2013). Understanding what drives exotic species richness and preventing introductions is the most efficient and cost-effective management option for protected areas (Tu, 2009). Around the globe, the number of exotic species in protected areas is a consequence of several interacting factors, such as the size and age of the park, native plant richness, and human density in surrounding areas (e.g., McKinney, 2006; White & Houlahan, 2007).

Biological invasions research has favored understanding plant introductions, especially in developed countries (Pyšek et al., 2008; Hulme et al., 2014). Yet in developing countries biodiversity threats are greatest and resources most limited (Myers et al., 2000), with research to understand and control exotic species in these countries much needed (Nuñez & Pauchard, 2010). At least 41 of the world’s 100 most invasive species are established in South America (IUCN, 2000), where many invasions are poorly studied or not considered as a priority among governments and citizens. Some exotic species are even favored and protected for their economic benefits to local communities (Lambertucci & Speziale, 2011; Speziale et al., 2012). Argentina is severely affected by the introduction of exotic species (Novaro, Funes & Walker, 2000; Anderson & Valenzuela, 2014); however, there are few broad scale reviews on this matter (e.g., Novillo & Ojeda, 2008; Merino, Carpinetti & Abba, 2009; Ballari, Anderson & Valenzuela, 2016). At least 402 exotic species are present in Argentina, of which 40%–50% have become invasive, causing considerable environmental damage (Brown et al., 2006). Our objective was to analyze how environmental, biotic, and anthropogenic factors influence exotic species richness within terrestrial protected areas in Argentina, and to compare across taxonomic groups to provide a basis for future research and management.

## Material and Methods

### Study area

The Argentinian National Parks Administration manages a system of national parks (hereafter NP), national reserves, national natural monuments, and marine parks co-managed with provincial governments. Argentina’s National Park System represents an array of protected areas of different sizes, urbanization levels, and climate. Together they comprise about 43,000 km^2^, (1.3% of the national territory), and are the protected areas with highest protection in the country: permanent monitoring and only tourism allowed in most areas (APN, 2007; Marinaro, Grau & Aráoz, 2012). The remaining protected areas (e.g., provincial parks, natural reserves) have little to no monitoring and allow high human disturbance (e.g., cattle grazing, forestry). Often, little or no biodiversity data are available (Brown et al., 2006), and part of them function as “paper parks” (Bertonatti & Giacchino, 2003), i.e., protected areas that have little or no formal management on the ground (Bonham, Sacayon & Tzi, 2008). The 44 terrestrial protected areas in the Argentinian National Parks System range from 0.08 to 7,270 km^2^, with variable adjacent urbanization and levels of human disturbance and diverse climates and biomes (SIB, 2014). A map of protected areas within the National Parks System can be accessed at http://www.sib.gov.ar/regiones (SIB, 2014).

### Data compilation

We compiled an initial list of 15 factors from 36 terrestrial protected areas (the areas excluded were data deficient). We obtained species lists and maps from the Biodiversity Information System (SIB, 2014) by the Argentinian National Parks Administration, which aims to collect, classify and organize biological data on protected areas under their jurisdiction. Data are evaluated and analyzed by technical staff before being entered into the species list for each area.

For each protected area, we collected the number of native and exotic species of plants, fishes, amphibians, reptiles, birds, and mammals (other taxa were data deficient). We also collected information on park age (years since designation), and park area (ha). Each area was assigned to one of six political regions predefined (SIB, 2014): Centro (Center), Centro-Este (East-center), Noreste (Northeast), Noroeste (Northwest), Patagonia Norte (North Patagonia) and Patagonia Austral (South Patagonia) (SIB, 2014). Using topographic maps (1:200,000) (SIB, 2014), we determined elevation range (100 m resolution), number of permanent rivers crossing protected area boundaries, and number of roads (all types) entering protected areas. We collected data on climate (1960–2014) from the National Weather Service (Servicio Meteorológico Nacional, 2015), which provides data on mean annual rainfall and mean annual temperature at national scale. Weather data was extracted from a national map divided into areas showing different mean values. Mean annual temperature ranged from 4 to 24 °C, and mean annual rainfall ranged from 25 to 1,800 mm. Mean annual number of visitors during 2003–2011 was obtained from an annual government report from the Resource Management Division of the National Parks Administration (APN, 2012). The Human Influence Index (hereafter HII) is a global dataset of 1-kilometer grid cells, combining population pressure, land use and infrastructure, and transportation access (WCS and CIESIN, 2005). We extracted the mean HII value and HII range (1 km res.) within each protected area; and the same parameters for a 20 km buffer zone around park borders representing surrounding disturbance (Spear et al., 2013).

### Analyses

We performed Spearman ranked correlations to assess collinearity of predictor variables (|*r*| > 0.70; Dormann et al., 2013). We used generalized linear models (GLMs) in software R (R Core Team, 2015) using package lme4 (Bates et al., 2015) and fit models using a Poisson distribution or negative binomial distribution for models with high overdispersion (i.e., ĉ > 1.0; Quinn & Keough, 2002). We tested model fit for both distributions using a *X*^2^ goodness of fit test (*α* = 0.05). Each park was our sample unit, and we specified exotic plant, exotic mammal, and exotic bird richness as the response variable for each model set, omitting fishes, reptiles, and amphibians from analyses due to the low number of exotic records.

Factors used to address the rich get richer hypothesis and the community richness hypothesis were number of native plants (for all taxa), number of native mammals (for exotic mammals), and number of native birds (for exotic birds). For the human disturbance hypothesis, factors included park age, number of roads entering area, mean number of visitors per year, and mean and range HII within and around the protected area. For the environmental hypothesis, factors included elevation range, park area, mean annual temperature, mean annual rainfall, number of rivers crossing area boundaries, and region.

We selected factors for each taxon-specific model by regression of each taxon on individual factors (univariate models). We then combined significant (*α* < 0.05) factors into a model set for each taxon. Since each factor may independently influence species richness, we tested all additive combinations of selected factors within each taxon as main effects. Factors were centered (mean = 0) and scaled (standard deviation = 1) before analyses to allow equal weight among factors measured on different scales. We used sample size corrected Akaike Information Criterion (AIC_c_) to rank model support for each taxon, and considered models competing if within 2 AIC_c_ units of the most parsimonious model (Burnham & Anderson, 2002). The negative binomial distribution does not allow for model averaging; therefore we selected the simplest top ranked model as the best fit (e.g.,  Spear et al., 2013). Choosing the simplest model within nested top models is a conservative approach appropriate to avoid overfitting (Richards, Whittingham & Stephens, 2011). Parameter estimates were considered significant if *α* < 0.05. Finally, we calculated the amount of deviance explained adjusted for the number of observations and parameters for each competing model (adj. D^2^; Guisan & Zimmermann, 2000) using package modEvA (Barbosa et al., 2015).

## Results

We compiled 22,963 native and 1,688 exotic records in Argentina’s National Parks System. Records of exotic species were 83% plants and 17% animals (9.5% mammals, 5.5% birds, 1.5% fishes, 0% reptiles, and 0% amphibians). Among vertebrates, we found a strong skew towards exotic mammals and birds. Due to the very low number of exotic occurrences, we omitted fishes, reptiles, and amphibians from subsequent analyses. There were no highly correlated factors (|*r*| > 0.70).

**Figure 1:**
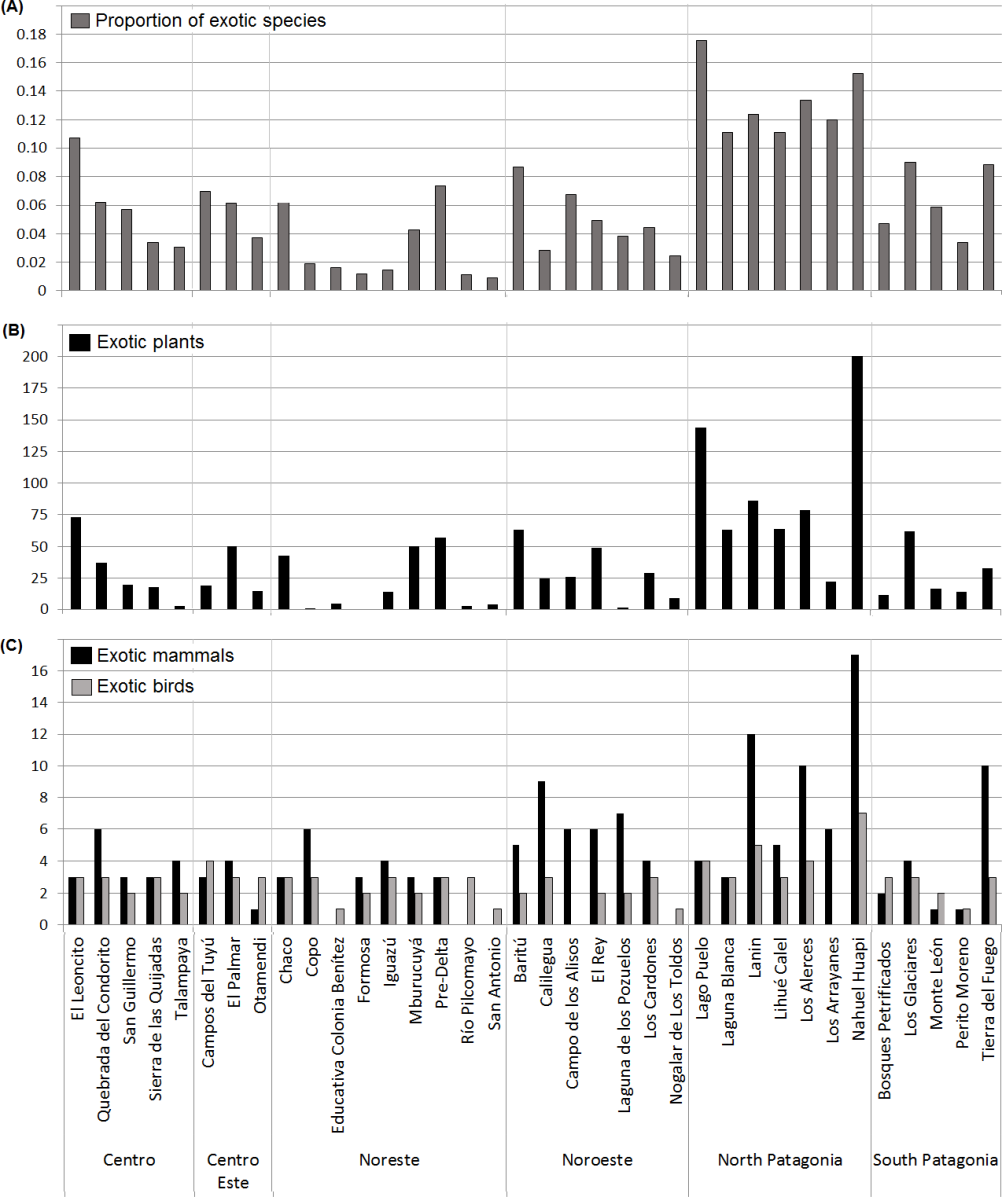
Exotic species documented in Argentina’s National Parks System by protected area. (A) Proportion of exotic species for all taxa combined, (B) number of exotic plants, and (C) number of exotic mammals and birds. Parks are categorized by political region: Center (Centro), East Center (Centro-Este), Northeast (Noreste), Northwest (Noroeste), North Patagonia (Patagonia Norte) and South Patagonia (Patagonia Austral).

The protected areas with most documented exotic species were Nahuel Huapi NP (227), Lago Puelo NP (152), Lanin NP (107), and Los Alerces NP (97), all within the North Patagonian region (Fig. 1). The protected areas with fewest documented exotic species were San Antonio Reserve (five), Formosa Reserve (five), Rio Pilcomayo NP (eight), and Tampalaya NP (nine), all in northern Argentina. Most frequently documented exotic plant families included grasses (Poaceae) present in 33 of 36 areas (91%), composites (Asteraceae) in 30 (83%), pink family (Caryophyllaceae) in 25 (75%), knotweed family (Polygonaceae) in 26 (70%), legumes (Fabaceae) in 25 (70%), and the rose family (Rosaceae) in 19 (52%). The most frequently reported exotic mammals were lagomorphs (28 areas; 78%); specifically, the European hare (*Lepus europaeus*) was present in 27 (75%). Following were domestic ungulates (e.g., cows, horses, and sheep) present in 21 (57%), and rodents (*Rattus* sp. and *Mus musculus*) listed in 11 areas (35%). Wild boar (*Sus scrofa*) was documented in 11 areas (35%), and exotic cervids (e.g., *Axis axis*, *Dama dama, Cervus elaphus*) in eight (22%). For carnivores, feral dogs (*Canis familiaris*) were reported in 11 areas (35%), American mink (*Neovison vison*) in six (17%), and feral cats (*Felis catus*) in four (11%). The most frequent documented exotic birds were the house sparrow (*Passer domesticus*) in 29 (78%), and the rock dove (*Columba livia*) in 25 (67%). The cattle egret (*Bubulcus ibis*) is widespread in Argentina’s protected areas (present in 33 areas [89%]), and while it is considered an exotic species by the National Parks Administration (SIB, 2014) we did not include it in subsequent analyses because it was not introduced by humans.

### Exotic plants

Univariate models used for selection of variables in the global model for exotic plant richness showed a positive influence of native plant richness (*p* < 0.001), park area (*p* = 0.034), elevation range (*p* = 0.003), rivers (*p* = 0.009), region (only North Patagonia, *p* = 0.023), park age (*p* = 0.020), and range HII (*p* = 0.017), and a negative influence of average temperature (*p* = 0.002). Variables present in competing models included native plant richness, mean temperature, and park area (Table 1). The top model suggested exotic plant richness increased with native plant richness, and decreased with increasing average temperature (Table 2). Park area (*β* =  − 0.18, *SE* = 0.15) was included with mean temperature and native plant richness in the only competing model, but was not significant (*p* = 0.24).

**Table 1:** Top models (ΔAICc ≤ 2.00) of the most influential predictors on exotic plant, mammal, and bird species richness in Argentina’s National Parks System. Number of parameters (*K*), log likelihood (LL), Akaike Information Criterion adjusted for small sample sizes value difference with top model (ΔAICc), Akaike model weights (*w*), and adjusted deviance explained (Adj. $D\hat {}2$ [%]) provided for each model.

Response	Predictors	*K*	LL	ΔAICc	*w*	Adj. $D\hat {}2$ (%)
Exotic plants	Temp + native plants	4	−154.77	0.00	0.68	45.00
	Temp + native plants + area	5	−154.14	1.46	0.33	45.03
Exotic mammals	Mean HII + range HII	3	−80.03	0.00	0.32	42.72
	Mean HII + range HII + elevation	4	−78.83	0.16	0.30	43.51
	Mean HII + range HII + native plants	4	−79.11	0.71	0.23	42.91
	Mean HII + range HII + rivers	4	−79.54	1.56	0.15	41.99
Exotic birds	Range HII	2	−56.59	0.00	0.51	36.54
	Range HII + roads	3	−56.04	1.28	0.27	39.04
	Range HII + rivers	3	−56.19	1.58	0.23	37.83

**Table 2:** Beta coefficients and their standard errors (SE) of the most influential predictors explaining the number of exotic plants, mammals, and birds in Argentina’s National Parks System.

	Exotic plants	Exotic mammals	Exotic birds
	*β* (SE)	*β* (SE)	*β* (SE)
No. native plants	0.67 (0.12)^*^		
Mean temperature	−0.35 (0.13)^*^		
Mean HII		−0.29 (0.10)^*^	
Range HII		0.42 (0.06)^*^	0.29 (0.08)^*^
Distribution	Negative binomial	Poisson	Poisson

**Notes.**

* denotes predictors significant at *α* < 0.05.

### Exotic mammals

Univariate models used for selection of variables in the global model for exotic mammal richness showed a positive influence of native plant richness (*p* = 0.001), park area (*p* = 0.001), elevation range (*p* = 0.001), rivers (*p* = 0.004), region (only North Patagonia, *p* = 0.034), park age (*p* = 0.011), roads (*p* = 0.017), and range HII (*p* < 0.001), and a negative influence of average temperature (*p* = 0.017), and mean HII (*p* = 0.045). Variables present in competing models included Mean HII, Range HII, elevation, native plants and rivers (Table 1). The top model for exotic mammal richness indicated that richness increased with Range HII and decreased with increased Mean HII; Range HII was the most influential factor (Table 2). Competing models also included Range HII and Mean HII, in addition to elevation range (*p* = 0.11), native plant richness (*p* = 0.18), or rivers (*p* = 0.32).

### Exotic birds

Univariate models used for selection of variables in the global model for exotic bird richness showed a positive influence of native plant richness (*p* = 0.02), park area (*p* = 0.009), rivers (*p* = 0.012), roads (*p* = 0.006), range HII (*p* < 0.001) and mean HII (*p* = 0.007). Variables present in competing models for exotic bird richness included Range HII, roads and rivers (Table 1). The top model included Range HII and indicated exotic bird richness increased with Range HII (Table 2). Competing models also included Range HII, but included either roads (*β* = 0.12, *SE* = 0.11, *p* = 0.287) or rivers (*β* = 0.10, *SE* = 0.10, *p* = 0.361).

## Discussion

In Argentina’s national park system, exotic plant richness was mostly influenced by native plant richness, supporting the rich get richer hypothesis, while exotic mammal and bird richness was driven mostly by anthropogenic factors, supporting the human disturbance hypothesis. The community richness hypothesis was not supported; no exotic taxon was negatively influenced by the number of native species. The factors with a consistent positive influence across taxa could be related, although indirectly, to an increase in spatial heterogeneity, either natural (native plant richness, e.g., Deutschewitz et al., 2003) or anthropogenic (spatial range of human disturbance). This supports that niche space variability and opportunities for colonization may facilitate establishment, in alignment with the “niche opportunity” concept (Shea & Chesson, 2002). We found that neither the mean nor range index of human disturbance in areas surrounding protected areas influenced exotic richness. In contrast, human population surrounding protected areas in South Africa was the best predictor of exotic plants and animals (Lodge, 1993).

We identified several exotic species that have become invasive in Argentina (e.g., wild boar, European hare, thistles *Cardus sp.,* wire grass *Cynodon dactylon*) that appear common within the national parks system. A national assessment of invasive mammals in Argentina found that half of the highest risk species were feral domestic animals (Lizarralde, 2016); in this study we found feral ungulates to be remarkably widespread in protected areas, followed by feral dogs in one third of the areas.

Since survey and research efforts vary at different regions and areas, data are likely of heterogeneous quality. Broad scale species lists suffer from inevitable flaws, such as imperfect detection (Royle, Nichols & Kery, 2005), and usually underestimate species richness numbers (McGeoch et al., 2012). However, national systematic species surveys are expensive and time consuming, constraining conservation planners to make best use of incomplete data (Braunisch & Suchant, 2010), particularly in countries with limited resources for management actions. In addition, we analyzed current exotic species richness patterns under the assumption that such richness can be explained by current or recent conditions. Worldwide, invasion patterns across taxa are temporally dynamic and the increase in numbers of exotic species does not show any sign of saturation (Seebens et al., 2017). Despite the expected limitations of the data, 36–45% of the deviance (i.e., lack of fit) was explained by our models, representing considerable support for observed relationships.

### Exotic plants

Most exotic species were plants, as found for protected areas in other countries (e.g., Spear et al., 2013). The most frequently reported exotic plants were grasses (e.g., *Lolium multiflorum*, *Cynodon dactylon*) and other flowering plant species (e.g., *Anthemis cotula, Cardus sp, Taraxacum officinale*) that are highly invasive and benefited by disturbance (IUCN, 2000). Exotic plants are commonly introduced through anthropogenic activities such as agriculture, transportation, or touristic activities and infrastructure (Kolar & Lodge, 2001; Pickering & Hill, 2007), and disturbance is an important driver of plant spread (Christen & Matlack, 2009), particularly for exotic grasses (Veldman & Putz, 2010). Yet, none of the anthropogenic variables we analyzed were strongly supported as pathways for exotic plants; it is possible we did not include variables that represented the human activities more directly related to plant introductions in our system.

Parks with greater native plant species richness were not more resistant to successful introductions; on the contrary, exotic plants were positively related to native plant richness supporting the rich get richer hypothesis (Stohlgren, Barnett & Kartesz, 2003). Similarly, areas with high native plant richness contained more exotic plants in USA (Stohlgren et al., 2008) and Argentina (Speziale & Ezcurra, 2011). Native and exotic species should have similar responses to broad environmental conditions (Levine & D’Antonio, 1999); being indirectly, but positively, correlated to habitat heterogeneity and increasing available resources (Davies, 2009). However, the exotic vs. native richness relationship appears dependent on spatial scale; being positively associated at large scales and negatively at smaller scales (Shea & Chesson, 2002; Chen et al., 2010).

Exotic plant richness was lower in warmer protected areas. Temperature positively influenced exotic plant richness in some European reserves (Pyšek, Jarosik & Kucera, 2002), but had no effect in US protected areas (McKinney, 2002; Allen, Brown & Stohlgren, 2009). Warmer temperatures may improve the likelihood of species establishing and reproducing successfully (Le Roux et al., 2013), and a regional study of exotic plants within Patagonia concluded warmer temperatures likely benefited invaders (Speziale & Ezcurra, 2011). However, exotic species richness was greater in the North Patagonia region, despite lower mean temperatures relative to other regions. This unexpected relationship may be explained by increased pathways for introductions from high outdoor recreation and different management policies (See ‘Regional Differences’ below). For example, even Patagonian parks located in remote mountain areas contain exotic plants, potentially because of their popularity as mountaineering destinations (Barros & Pickering, 2014).

Larger areas are usually associated with greater environmental heterogeneity (Wiens, 1989), and may provide more opportunities for colonizing species (MacArthur & Wilson, 1963). However, in our system, park area was not influential on exotic plant richness. Similarly, support from other studies has been very limited, with effects of area on number of exotic species ranging from non-influential (Pyšek, Jarosik & Kucera, 2002; Allen, Brown & Stohlgren, 2009) to influential but having a negligible effect (McKinney, 2002).

### Exotic mammals

The range of human disturbance within each park was the most influential factor on exotic mammal richness. Anthropogenic influence, represented mostly as disturbance and propagule pressure, has often been associated with exotic species richness (e.g., Chown, Hull & Gaston, 2005; Le Roux et al., 2012), including in protected areas (McKinney, 2006; Spear et al., 2013). The links between human activities and mammal introductions around the world are abundant (e.g., livestock, companions, commensals, or sports; Clout & Russell, 2008). In our study, most exotic mammals belong to species usually introduced for food (e.g., cows, sheep), hunting (e.g., lagomorphs, ungulates), or are associated with human-modified areas (e.g., rodents). This indicates that direct human actions, either by intentional introductions or urban expansion, are the main causes increasing exotic mammal richness in our study area, although successful establishment may be dependent on other conditions (e.g., climate, Clout & Russell, 2008). Even though the most influential factor was the range of human disturbance (with a positive effect), the mean human influence value had an opposite relationship with exotic mammal richness. We propose this unexpected relationship is a result of large and remote parks, mostly in North Patagonia, having low mean disturbance values, but high range disturbance values, representing that the parks had large sections with low disturbance, but they also included smaller sections with very high human disturbance that could create pathways for introductions.

Elevation range, native plant richness, and number of intersecting rivers did not influence exotic mammal richness. Range in elevation can represent habitat heterogeneity and extreme climatic environments, and had a positive association with exotic plant richness among US national parks (Allen, Brown & Stohlgren, 2009). Elevation remains a scarcely studied factor in relation to exotic species, possibly because large scale variation in elevation may not sufficiently describe small scale habitat variability (Stohlgren et al., 2005). On the other hand, native plant richness is usually correlated to increased heterogeneity and resources (Davis, Grime & Thompson, 2000), and a US study of protected areas found that it had a positive effect on exotic mammal richness (McKinney, 2006), but its influence on exotic animals in this study was limited. Finally, rivers and riparian areas can serve as corridors for mammals and plants (Naiman, Decamps & Pollock, 1993; Less & Peres, 2008) but were of limited influence in our system; other studies have found mixed results (Allen, Brown & Stohlgren, 2009; Spear et al., 2013).

### Exotic birds

As with exotic mammals, range of human disturbance had the greatest influence on exotic bird richness. Globally, exotic bird establishment appears to be influenced by the degree of environmental similarity between the native range and introduction site (Blackburn & Duncan, 2001); though Carrete & Tella (2008) suggested the most successful exotic bird species are those caught in the wild and traded on the pet market. However, most exotic bird species we documented were not pets, but human-associated birds (e.g., rock dove and house sparrow), likely because urbanized birds have high establishment success in novel environments (Moller et al., 2015).

Riparian corridors can facilitate movement of some bird species (Gillies & Clair, 2008), and the amount of road networks and their proximity to natural communities potentially make roads important pathways of invasion (Forman et al., 2003). However, roads and rivers were not influential on exotic bird richness in our study, their influence may have been unimportant compared to the range of combined anthropogenic disturbance. Likewise, in South Africa roads were found to be non-influential on exotic richness (Spear et al., 2013).

### Regional Differences

We observed greater richness of exotic plants and mammals in North Patagonia, and the five parks with most invasive species (all taxa combined) were located in this region (Fig. 1). These parks are proximate to cities that are important tourist destinations, offering many outdoor activities within protected areas that likely serve as pathways for exotic species introductions (Barros & Pickering, 2014). The policies applied to Patagonia protected areas during the 20th century were eclectic: the objective of wilderness areas conservation was followed, while initiatives to sell areas within the national parks and introduce exotic species were permitted, even though this model was later rejected (Vejsbjerg, Núñez & Matossian, 2014). Consequently, many species have been introduced for forestry (e.g., conifers), fishing (e.g., salmonids), and hunting (e.g., European hare, wild boar, red deer) (APN, 2007; Bonino Vasallo, 2010).

## Conclusions

Exotic species introductions and invasions are widespread and protected areas are susceptible to them, demonstrating that environmental protection is usually less effective than desired (Pressey, Visconti & Ferraro, 2015), particularly in developing countries (Bonham, Sacayon & Tzi, 2008). Identifying patterns of exotic species richness is a first step to develop conservation strategies, particularly when resources are limited. We found that areas of high native richness and human disturbance heterogeneity appear more vulnerable to exotic species establishment, therefore restricting human settlements or activities (Foxcroft et al., 2011; Anderson et al., 2015) may limit the impacts of human disturbance. Protected areas with greater range in human disturbance will face continuing pressure, complicating management efforts. Limiting the use of exotic species for commercial or recreational use within and adjacent to protected areas will likely facilitate control of invasions (Ballari, Anderson & Valenzuela, 2016; Lambertucci & Speziale, 2011). Our inferences and interpretations are limited by availability and standardization of exotic species data collected among Argentina’s national parks. Regional or taxa-specific reviews on exotic species management actions have been recently published (Sanguinetti et al., 2014; Lizarralde, 2016) which emphasize the lack of information and effective legislation. It is imperative to allocate resources for comprehensive and systematic surveys of native and exotic species richness (McGeoch et al., 2012), as well as the area they occupy, within and outside all protected areas (not only national parks) in Argentina. This additional data will help to further disentangle the patterns and mechanisms of exotic species richness and distribution, serving to prioritize protected areas with greatest need of monitoring and conservation, and identify which actions would be most helpful.

##  Supplemental Information

10.7717/peerj.5514/supp-1 Supplemental Information 1 Argentina’s national parks native and exotic richness, and environmental and anthropogenic variables

## Additional Information and Declarations

### Data Availability

The following information was supplied regarding data availability: The raw data are provided in a Supplemental File.
